# Efficacy of balloon guide catheter in endovascular treatment according to occlusion location

**DOI:** 10.3389/fneur.2026.1867575

**Published:** 2026-09-30

**Authors:** Hicret Betul Adiyaman, Zehra Uysal Kocabaş, Ozlem Aykac, Atilla Ozcan Ozdemir

**Affiliations:** 1 Department of Neurology, Sivas Numune Hospital, Ministry of Health, Sivas, Türkiye; 2 Department of Neurology, Faculty of Medicine, Eskisehir Osmangazi University, Eskisehir, Türkiye

**Keywords:** acute ischemic stroke, balloon guide catheter (BGC), distal embolism, first-pass effect, large vessel occlusion, mechanical thrombectomy, recanalization

## Abstract

**Background and purpose:**

The clinical and procedural benefits of balloon guide catheter (BGC) use during mechanical thrombectomy remain debated, and whether these associations vary by occlusion location is uncertain. We evaluated the association of BGC use with thrombectomy outcomes and examined potential effect modification by occlusion location.

**Methods:**

We retrospectively analyzed 475 patients with anterior-circulation large-vessel occlusion undergoing mechanical thrombectomy (BGC, *n*=242; non-BGC, *n*=233). Procedural, angiographic, and clinical outcomes were compared between groups. Multivariable logistic regression models adjusted for age, sex, baseline NIHSS, collateral status, occlusion location, atrial fibrillation, hypertension, diabetes mellitus, and coronary artery disease. BGC × occlusion-location interactions were assessed using adjusted generalized linear models.

**Results:**

BGC use was independently associated with higher odds of 90-day functional independence (mRS 0–2; adjusted OR [aOR], 1.61; 95% CI, 1.05–2.48; *p*=0.029) and lower odds of embolization to new territory (aOR, 0.53; 95% CI, 0.35–0.81; *p*=0.004). BGC use was also associated with higher odds of vasospasm (aOR, 1.68; 95% CI, 1.09–2.58; *p*=0.018), whereas associations with first-pass recanalization and hemorrhagic complications were not significant. No significant BGC × occlusion-location interaction was observed for any evaluated outcome (all P for interaction>0.05).

**Conclusion:**

BGC use was independently associated with favorable 90-day functional outcome and reduced embolization to new territory, but with increased vasospasm. No evidence of effect modification by occlusion location was identified. These findings support potential overall benefits of BGC use while emphasizing that location-specific effects remain unproven and require prospective evaluation.

## 1 Introduction

Mechanical thrombectomy (MT) has become the standard treatment for acute ischemic stroke (AIS) caused by large vessel occlusion (LVO), following multiple randomized controlled trials demonstrating its superiority over best medical therapy alone in appropriately selected patients (1, 2). As endovascular techniques continue to evolve, increasing attention has focused on procedural strategies that may further improve reperfusion quality, procedural efficiency, and clinical outcomes.

Among these adjunctive strategies, the balloon guide catheter (BGC) has been widely adopted during stent retriever thrombectomy. Inflation of the balloon temporarily arrests or reverses antegrade blood flow, while simultaneous aspiration through the guide catheter may reduce distal thrombus migration during clot retrieval. These mechanisms are thought to decrease thrombus fragmentation, limit distal embolization, and facilitate more effective clot extraction (3, 4).

Several observational studies, registry analyses, and meta-analyses have suggested that BGC use may be associated with higher first-pass recanalization rates, improved angiographic reperfusion, shorter procedure times, and better functional outcomes compared with conventional guide catheters (5–8). Likewise, imaging studies have reported reduced numbers of peripheral emboli following thrombectomy performed with BGC assistance (7). However, most available evidence originates from retrospective observational studies that are inherently susceptible to selection bias and confounding. Moreover, recent randomized evidence, including the PROTECT-MT trial, has produced less consistent findings, suggesting that the clinical benefit of BGC may not be uniform across all patients and procedural settings (9). These observations indicate that the effectiveness of BGC may depend on anatomical and procedural factors that have not yet been fully clarified.

One potential explanation for these heterogeneous findings is that the effect of BGC may vary according to occlusion location. Differences in vessel caliber, clot burden, vascular anatomy, collateral circulation, and the technical characteristics of thrombectomy among internal carotid artery (ICA), proximal middle cerebral artery (MCA-M1), and distal middle cerebral artery (MCA-M2) occlusions may influence the degree to which proximal flow arrest and aspiration provide procedural advantage. Despite this biologically plausible hypothesis, relatively few studies have specifically investigated whether the association between BGC use and procedural or clinical outcomes differs according to occlusion location, and the available evidence remains limited.

Therefore, the present study aimed to compare angiographic, procedural, and clinical outcomes of MT performed with and without BGC after stratification by occlusion site. We hypothesized that the magnitude of benefit associated with BGC use is not uniform across ICA, MCA-M1, and MCA-M2 occlusions and that occlusion location may modify the relationship between BGC use and thrombectomy outcomes.

## 2 Materials and methods

### 2.1 Study design and patient population

This was a retrospective analysis of prospectively collected data from consecutive patients who underwent endovascular treatment for anterior circulation AIS-LVO at Eskisehir Osmangazi University Medical Faculty Hospital between May 2015 and December 2024. The institutional ethics committee approved the study protocol.

Inclusion criteria were: (1) age ≥18 years; (2) symptom onset within 6 h; (3) baseline NIHSS score ≥5; (4) pre-stroke mRS score ≤2; and (5) angiographically confirmed occlusion of the ICA, MCA-M1, or MCA-M2 segment. Exclusion criteria were posterior circulation occlusion and a symptom-to-treatment interval exceeding 6 h.

Patients were classified into two main groups: those treated with BGC and those without BGC. Each group was further stratified by occlusion location: proximal MCA (MCA-M1), distal MCA (MCA-M2), and ICA.

### 2.2 Endovascular procedure

All procedures were performed at a single comprehensive stroke center by an experienced neurointerventional team using standardized institutional treatment protocols and under conscious sedation or general anesthesia using a Siemens Artis Zee monoplane neuroangiography system. The choice of BGC, thrombectomy technique, and devices was left to the operator’s discretion. Three first-line techniques were recorded: isolated stent retriever (with or without BGC), aspiration-first (ADAPT), and combined (BGC + distal access catheter [DAC] + stent retriever). BGC devices used included the Cello (14.9%), Concentric (58.7%), and Flowgate (26.4%) catheters in 8F (46.3%) and 9F (53.7%) configurations, positioned at the upper (74.8%) or lower (25.2%) cervical ICA.

### 2.3 Outcome measures

The primary angiographic outcome was embolization into a previously uninvolved vascular territory (embolization to new territory, ENT), as identified on the final angiographic runs. Secondary angiographic outcomes included first-pass recanalization (FPR) (defined as thrombolysis in cerebral infarction (mTICI) 2c–3 after a single device pass), vasospasm, new territory embolism and hemorrhagic complications. Hemorrhagic events were assessed on follow-up brain imaging and classified according to the European Cooperative Acute Stroke Study (ECASS) criteria. For statistical analyses, hemorrhagic complications were evaluated as the presence or absence of any intracranial hemorrhage. Clinical outcomes included discharge mRS and 90-day mRS, with functional independence defined as mRS 0–2. Procedural variables included the number of intracranial passes and puncture-to-recanalization time.

#### 2.3.1 Outcome adjudication

Angiographic outcomes, including the final modified Thrombolysis in Cerebral Infarction (mTICI) grade, first-pass effect (FPE), and embolization to new territory (ENT), were assessed by the treating neurointerventional operator based on the procedural digital subtraction angiography images. Clinical outcomes were assessed independently by neurologists during routine outpatient follow-up. Functional outcome at 90 days was evaluated using the modified Rankin Scale (mRS).

### 2.4 Statıstıcal analysıs

Categorical variables were compared using the Chi-square test or Fisher’s exact test, and continuous variables using the Mann–Whitney *U* test. Descriptive statistics are presented as mean ± standard deviation or frequency and percentage.

Covariates were selected based on their clinical relevance as potential confounders of the association between BGC use and thrombectomy outcomes, as well as observed baseline differences between the treatment groups, rather than solely on univariable statistical significance. Age, sex, baseline NIHSS score, and collateral status were included as clinically relevant determinants of stroke outcome and procedural success, while occlusion location was included because of its potential relationship with both BGC selection and thrombectomy outcomes. Atrial fibrillation, hypertension, type 2 diabetes mellitus, and coronary artery disease were included to account for differences in vascular risk and comorbidity burden between the treatment groups. The same core covariate set was retained across the procedural (first-pass recanalization and embolization to new territory), clinical (90-day functional independence), and safety (hemorrhagic complications and vasospasm) models to provide consistent adjustment across outcomes and to avoid outcome-driven covariate selection. Although the relative importance of individual covariates may differ across procedural, clinical, and safety outcomes, a common adjustment set was intentionally used because these variables could plausibly influence treatment selection and/or the respective outcomes, while maintaining comparability across models.

Multivariable binary logistic regression models were constructed for first-pass recanalization, 90-day functional independence, embolization to new territory, hemorrhagic complications, and vasospasm. A common adjustment set was used across all multivariable models to estimate the association between BGC use and procedural, clinical, and safety outcomes under a consistent confounding structure. Each model included BGC use, occlusion location, age, sex, baseline NIHSS score, collateral status, atrial fibrillation, hypertension, type 2 diabetes mellitus, and coronary artery disease. These variables were selected as baseline demographic, clinical, anatomical, and comorbidity characteristics that could contribute to differences between the BGC and non-BGC groups. Covariates were not selected or removed according to univariable statistical significance. The same adjustment set was retained across outcomes to maintain comparability between models and to avoid outcome-dependent variable selection. Adjusted odds ratios (aORs) with 95% confidence intervals (CIs) were reported.

Missing data were handled using complete-case analysis, and no imputation was performed. Participants with missing data for the outcome or any covariate included in the model were excluded from the analysis. All multivariable models included 473 of 475 patients; 2 patients (0.4%) were excluded because of missing data.

To formally assess whether the association between BGC use and study outcomes differed according to occlusion location, multivariable generalized linear models with a binomial distribution and logit link function were constructed for the same binary outcomes. Each interaction model included BGC use, occlusion location, age, sex, baseline NIHSS score, collateral status, atrial fibrillation, hypertension, type 2 diabetes mellitus, coronary artery disease, and the BGC × occlusion-location interaction term. Interaction effects were evaluated using Type III Wald chi-square statistics.

Model diagnostics included assessment of multicollinearity using tolerance and variance inflation factor (VIF) values and assessment of model calibration using the Hosmer–Lemeshow goodness-of-fit test. No evidence of problematic multicollinearity was identified (VIF range, 1.021–1.257; tolerance range, 0.796–0.979). Hosmer–Lemeshow tests showed no evidence of poor calibration across the multivariable models (all *p* > 0.05). A two-sided *p* value <0.05 was considered statistically significant. All analyses were performed using SPSS Statistics version 22.0 (IBM Corp., Chicago, IL, United States).

## 3 Results

A total of 859 patients with acute ischemic stroke were screened for eligibility between May 2015 and December 2024. Of these, 384 patients were excluded because of posterior circulation occlusion (*n* = 135), anterior cerebral artery occlusion (*n* = 20), absence of endovascular treatment (*n* = 25), baseline NIHSS score <5 (*n* = 66), or symptom onset >6 h (*n* = 138). The final study cohort comprised 475 patients, including 233 treated without a balloon guide catheter (BGC) and 242 treated with a BGC. The patient selection process is detailed in Figure 1.

**Figure 1:**
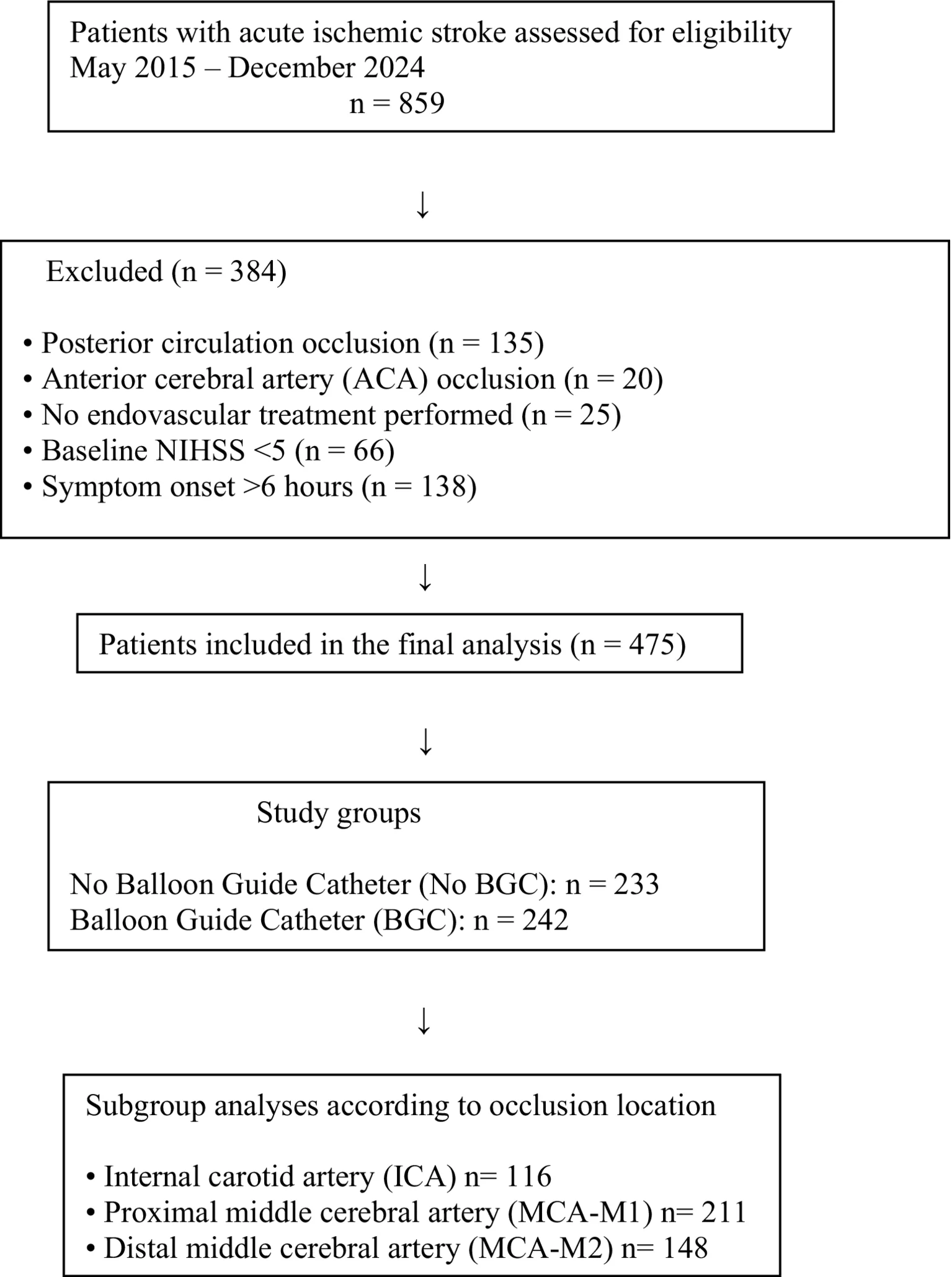
Patient selection flow diagram.

The most commonly used BGC size was 9F (53.7%), and the majority were positioned at the upper cervical ICA (74.8%). The Concentric catheter was the most frequently used device (58.7%). Baseline characteristics and catheter specifications are summarized in Tables 1, 2.

**Table 1:** Balloon guide catheter characteristics (BGC group only).

Characteristic	*n* (%)
Position	
Upper cervical	181 (74.8)
Lower cervical	61 (25.2)
Size	
8F	112 (46.3)
9F	130 (53.7)
Device	
Concentric	142 (58.7)
FlowGate	64 (26.4)
Cello	36 (14.9)

BGC, balloon guide catheter; F, French.

**Table 2:** Baseline characteristics by Balloon Guide Catheter use.

Variable	No BGC (*n* = 233)	BGC (*n* = 242)	*p* value
Female sex, *n* (%)	117 (50.2%)	103 (42.6%)	0.95
Age, years (mean ± SD)	68.7 ± 11	57.7 ± 14	**<0.001**
Admission NIHSS			
(mean ± SD)	15.2 ± 5	14.4 ± 5	0.74
Occlusion			
MCA M1	97(41.6%)	114 (47.1%)	0.442
MCA M2	78 (33.5%)	70 (28.4%)	
ICA	58 (24.9%)	58 (24%)	
ASPECTS (mean ± SD)	8,29 ± 1,74	8,89 ± 1,71	**<0.001**
Comorbidites			
Hypertension	164 (63.1%)	87 (44.6%)	**<0.001**
Atrial fibrillation	106 (42.2%)	48 (25.4%)	**<0.001**
Coronary artery disease	84 (32.2%)	46 (23.6%)	**0.044**
Type 2 diabetes mellitus	69 (26.4%)	40 (20.5%)	0.142
Current smoking	72 (27.6%)	64 (32.8%)	0.227
Obesity	42 (16.1%)	30 (15.4%)	0.838

BGC, balloon guide catheter; NIHSS, National Institutes of Health Stroke Scale; SD, standard deviation. ASPECTS, Alberta Stroke Program Early CT Score, MCA, middle cerebral artery, ICA, internal carotid artery.

A total of 475 patients were included: 242 in the BGC group and 233 in the non-BGC group. In the BGC group, 57.4% were male and 42.6% female. BGC-treated patients were significantly younger than non-BGC patients (mean age 57.7 ± 14 vs. 68.7 ± 11 years).

Compared with the non-BGC group, patients treated with BGC had a lower prevalence of atrial fibrillation (25.4% vs. 42.2%, *p* < 0.001), hypertension (44.6% vs. 63.1%, *p* < 0.001), and coronary artery disease (23.6% vs. 32.2%, *p* = 0.044). No significant between-group differences were observed in the prevalence of type 2 diabetes mellitus, obesity, or current smoking status. (Table 2).

The distribution of thrombectomy techniques differed significantly between the groups (*p* < 0.001). Combined techniques were more frequently used in the non-BGC group, whereas isolated stent-retriever thrombectomy was more common in the BGC group.

Angiographic reperfusion outcomes differed significantly between the two groups. Patients treated with a balloon guide catheter achieved complete reperfusion (mTICI 3) substantially more frequently than those treated without a balloon guide catheter (29.3% vs. 9.9%). Conversely, mTICI 2b reperfusion was more common in the non-BGC group (35.6% vs. 16.5%). Overall, the distribution of final mTICI grades differed significantly between groups (*χ*^2^ = 42.19, *p* < 0.001).

In contrast, first-pass recanalization rates were comparable between the BGC and non-BGC groups (51.7% vs. 47.6%, *p* = 0.382). No significant differences were observed in puncture-to-recanalization time (44.2 ± 29 vs. 45.3 ± 25 min, *p* = 0.140) or the total number of intracranial passes (2 ± 1 vs. 2 ± 1, *p* = 0.783).

Favorable functional outcome (mRS 0–2) was more frequent in the BGC group both at discharge (56.2% vs. 39.1%, *p* < 0.001) and at 90 days (65.7% vs. 46.8%, *p* < 0.001). Hemorrhagic complication rates were similar between groups (27.3% vs. 29.2%, *p* = 0.643). New territory embolism was less frequent in the BGC group (54.5% vs. 68.2%, *p* = 0.002), whereas vasospasm was more frequently observed in the BGC group (41.7% vs. 26.2%, *p* < 0.001) (Table 3).

**Table 3:** Procedural and clinical outcomes by balloon guide catheter use.

Variable	No BGC (*n* = 233)	BGC (*n* = 242)	*P* value
Technique *n* (%)			
Aspiration-first,	57 (24.5%)	73 (30.2%)	**<0.001**
Combined technique	156 (67%)	49 (20.2%)	
Isolated stent retriever	20 (8.6%)	120 (49.6%)	
TICI score *n* (%)			
mTICI 0–2a	29 (12.4%)	41 (16.9%)	**<0.001**
mTICI 2b	83 (35.6%)	40 (16.5%)	
mTICI 2c	98 (42.1%)	90 (37.2%)	
mTICI 3	23 (9.9%)	71 (29.3%)	
First pass recanalization	111 (47.6%)	125 (51.7%)	0.382
Puncture-to-recanalization time, min (mean ± SD)	45.3 ± 25	44.2 ± 29	0.140
Total intracranial passes (mean ± SD)	2 ± 1	2 ± 1	0.783
mRS 0–2 at discharge, *n* (%)	91 (39.1%)	136 (56.2%)	**<0.001**
mRS 0–2 at 90 days, *n* (%)	109 (46.8%)	159 (65.7%)	**<0.001**
Hemorrhagic complications	68 (29.2%)	66 (27.3%)	0.643
New territory embolism	159 (68.2%)	132 (54.5%)	**0.002**
Vasospasm	61 (26.2%)	101 (41.7%)	<0.001

In multivariable analysis, BGC use was independently associated with higher odds of 90-day functional independence (aOR, 1.61; 95% CI, 1.05–2.48; *p* = 0.029) and lower odds of embolization to new territory (aOR, 0.53; 95% CI, 0.35–0.81; *p* = 0.004). BGC use was also associated with higher odds of vasospasm (aOR, 1.68; 95% CI, 1.09–2.58; *p* = 0.018). In contrast, BGC use was not independently associated with first-pass recanalization (aOR, 1.35; 95% CI, 0.89–2.04; *p* = 0.155) or hemorrhagic complications (aOR, 1.15; 95% CI, 0.73–1.79; *p* = 0.544). (Table 4).

**Table 4:** Multivariable binary logistic regression analyses for study outcomes.

Outcome	*β*	SE	Adjusted OR	(95% CI)	*P* value
First-pass recanalization	0.300	0.211	1.35	(0.89–2.04)	0.155
Functional independence (90-day mRS 0–2)	0.478	0.219	1.61	(1.05–2.48)	**0.029**
Embolization to new territory	−0.623	0.215	0.53	(0.35–0.81)	**0.004**
Hemorrhagic complications	0.138	0.227	1.15	(0.73–1.79)	0.544
Vasospasm	0.519	0.220	1.68	(1.09–2.58)	**0.018**

OR, odds ratio; CI, confidence interval; NIHSS, National Institutes of Health Stroke Scale. Footnote: All models were adjusted for age, sex, baseline NIHSS score, collateral status, occlusion location, atrial fibrillation, hypertension, type 2 diabetes mellitus, and coronary artery disease. OR, Odds Ratio; CI, Confidence Interval; SE, Standard Error. Bold values indicate statistical significance (*p* < 0.05).

Full multivariable model results are provided in Supplementary Tables S1–S5.

No significant BGC × occlusion-location interaction was observed for first-pass recanalization (P for interaction = 0.244), 90-day functional independence (*p* = 0.280), embolization to new territory (*p* = 0.267), hemorrhagic complications (*p* = 0.579), or vasospasm (*p* = 0.063) (Table 5). Interaction effect estimates and 95% CIs are provided in the Supplementary Tables S6–S10.

**Table 5:** Interaction analyses between balloon guide catheter use and occlusion location.

Outcome	*P* for interaction
First-pass recanalization	0.244
90-day mRS 0–2	0.280
Embolization to new territory	0.267
Hemorrhagic complications	0.579
Vasospasm	0.063

NIHSS, National Institutes of Health Stroke Scale. Values represent *P* for interaction obtained from multivariable generalized linear models including balloon guide catheter use, occlusion location, the interaction term (BGC × occlusion location), Age, sex, baseline NIHSS, collateral status, atrial fibrillation, hypertension, diabetes mellitus, coronary artery disease.

As a sensitivity analysis, an ordinal logistic regression (shift analysis) across the full 90-day modified Rankin Scale distribution was performed. The proportional odds assumption was satisfied (Test of Parallel Lines, *p* = 0.276), and the overall model was significant (*χ*^2^ = 79.26, *p* < 0.001). After adjustment for age, sex, baseline NIHSS, collateral status, and occlusion location, balloon guide catheter use remained independently associated with a more favorable functional outcome (adjusted common OR 1.49, 95% CI 1.05–2.12; *p* = 0.026). Older age (OR 1.02 per year, 95% CI 1.01–1.03; *p* = 0.002) and higher baseline NIHSS (OR 1.09 per point, 95% CI 1.06–1.13; *p* < 0.001) were associated with worse functional outcomes, whereas good collateral status was associated with improved functional recovery (Supplementary Table S11).

## 4 Discussion

The present study evaluates the association between BGC use and procedural and clinical outcomes during mechanical thrombectomy for anterior circulation LVO, with particular attention to whether these associations vary according to occlusion location. Previous studies have explored this question using different methodological approaches. Kim et al. evaluated outcomes after contact aspiration thrombectomy according to both BGC use and occlusion location and reported location-dependent differences in angiographic and clinical outcomes (10). More recently, Knapen et al., using data from the MR CLEAN Registry, formally investigated heterogeneity of the association between BGC use and treatment outcomes by incorporating interaction analyses, providing a more robust framework for assessing potential effect modification (11). In the present study, we similarly evaluated BGC × occlusion-location interactions across multiple procedural, clinical, and safety outcomes while additionally accounting for relevant baseline characteristics and comorbidities. Although descriptive differences were observed across occlusion sites, none of the formal interaction tests reached statistical significance, indicating no evidence that the association between BGC use and the evaluated outcomes was significantly modified by occlusion location.

Our findings are consistent with previous *in vitro*, *in vivo*, and clinical evidence suggesting that BGC use may reduce embolization to new territory during mechanical thrombectomy (3–8). In our cohort, embolization to new territory was less frequent in patients treated with BGC than in those treated without BGC (54.5% vs. 68.2%, *P* = 0.002), and this association persisted after multivariable adjustment for relevant baseline characteristics and comorbidities (adjusted OR, 0.54; 95% CI, 0.35–0.82; *p* = 0.004). Experimental studies have suggested that proximal flow arrest and aspiration with BGC may reduce clot fragmentation during stent-retriever retrieval (4), while Schönfeld et al. demonstrated fewer subangiographic peripheral emboli on high-resolution DWI with BGC-assisted thrombectomy (7). Taken together, our findings add to the observational evidence supporting an association between BGC use and reduced embolization during thrombectomy, although the retrospective nature of our study precludes causal inference.

Previous observational studies have suggested that the association between BGC use and thrombectomy outcomes may vary according to occlusion location, although the available evidence remains inconclusive. Kim et al. reported numerically more favorable functional and angiographic outcomes with BGC use in some ICA-involving subgroups. (12). However, these findings were derived from relatively small subgroup samples and should be interpreted cautiously, particularly in the absence of formal interaction testing. Importantly, the lack of statistical significance in some MCA-only comparisons should not be interpreted as evidence of equivalence or absence of an association. Hansen et al. similarly reported variations in the association between BGC use and angiographic outcomes across procedural settings (13). Taken together, these studies and our findings demonstrate broadly consistent observational patterns across different cohorts and study designs, rather than independent replication of a location-specific treatment effect.

In our cohort, descriptive analyses likewise suggested variation in BGC-associated outcomes across occlusion locations; however, formal BGC × occlusion-location interaction analyses did not demonstrate significant effect modification for first-pass recanalization, 90-day functional independence, embolization to new territory, hemorrhagic complications, or vasospasm. These findings highlight the distinction between observing statistical significance within an individual subgroup and demonstrating a statistically significant difference in treatment effect between subgroups. Accordingly, our data do not establish that a particular occlusion location derives greater benefit from BGC use, and the location-specific patterns observed in retrospective studies should be considered hypothesis-generating.

From a mechanistic perspective, an influence of occlusion location on BGC effectiveness remains biologically plausible. In ICA terminus occlusions, the proximal location and larger thrombus burden may allow balloon inflation in the cervical ICA to produce more complete flow arrest and facilitate aspiration-assisted clot retrieval. In isolated MCA-M1 occlusions, collateral inflow through the anterior and posterior communicating arteries may partially preserve intracranial flow despite proximal ICA flow arrest, potentially attenuating the hemodynamic effect of balloon inflation. For more distal MCA-M2 occlusions, the greater distance between the balloon and thrombus, together with collateral inflow, may further reduce the influence of proximal flow control. Nevertheless, these mechanistic considerations remain hypothetical, as neither observational subgroup patterns nor our formal interaction analyses establish a location-specific treatment effect. Adequately powered prospective studies specifically designed to assess heterogeneity of BGC effects according to occlusion location are warranted.

In the unadjusted analysis, functional independence at 90 days was more frequent among BGC-treated patients, including a marked numerical difference within the MCA-M1 subgroup. However, the BGC group was substantially younger and differed in several baseline characteristics, raising the possibility that the subgroup-level difference in functional outcome was influenced by confounding. In the overall cohort, the association between BGC use and 90-day functional independence persisted after adjustment for age, sex, baseline NIHSS, collateral status, occlusion location, atrial fibrillation, hypertension, diabetes mellitus, and coronary artery disease (adjusted OR, 1.61; 95% CI, 1.05–2.48; *p* = 0.029). Nevertheless, formal interaction testing did not demonstrate that this association differed significantly according to occlusion location (P for interaction = 0.280). Therefore, while the adjusted overall analysis supports an independent association between BGC use and favorable 90-day functional outcome, our data do not provide sufficient evidence to attribute a greater clinical benefit specifically to MCA-M1 occlusions.

Final angiographic reperfusion also differed between the BGC and non-BGC groups in our cohort, with complete reperfusion (mTICI 3) observed more frequently in BGC-treated patients (29.3% vs. 9.9%), although these unadjusted differences should be interpreted in the context of baseline and procedural imbalances between groups. Previous imaging studies have suggested that BGC use may also influence embolic burden beyond that captured by conventional angiographic reperfusion grading. Schönfeld et al. (7) reported fewer peripheral embolic lesions on high-resolution DWI among patients undergoing BGC-assisted thrombectomy.

However, that study included only patients with successful reperfusion and had a very small BGC-treated sample, introducing potential selection bias and limiting the precision and generalizability of the findings. Moreover, the observed differences in peripheral embolic burden were not accompanied by demonstrable differences in functional outcome. Therefore, although subangiographic embolization represents a biologically plausible mechanism through which proximal flow control might influence tissue-level outcomes, current evidence is insufficient to establish that reductions in such emboli mediate improved functional outcome after BGC-assisted thrombectomy (7).

## 5 Limitations

Several limitations should be acknowledged. First, the retrospective, single-center design introduces potential selection bias and residual confounding. Although multivariable models were adjusted for major baseline characteristics and available comorbidities, unmeasured factors such as vascular anatomy and clot characteristics or etiology could not be fully accounted for. Second, the study spans nearly a decade, during which thrombectomy devices, procedural strategies, and operator experience evolved; because treatment year was not available as an analyzable variable, temporal confounding could not be assessed. Third, although all patients had a pre-stroke mRS of 0–2, individual baseline mRS categories were unavailable, precluding adjustment for pre-stroke functional status. Angiographic outcomes were also assessed by the treating team rather than an independent core laboratory, and procedural decisions were influenced by operator preference. Finally, subgroup findings should be considered exploratory, as formal BGC × occlusion-location interaction testing did not demonstrate significant effect modification and the study may have been underpowered to detect modest interaction effects.

## 6 Conclusion

Overall, our findings suggest that BGC use may be associated with selected clinical and procedural outcomes after mechanical thrombectomy, but they do not provide evidence of a differential treatment effect according to occlusion location. After adjustment for relevant baseline characteristics and comorbidities, BGC use remained independently associated with higher odds of 90-day functional independence and lower odds of embolization to new territory, while no independent association was observed with first-pass recanalization or hemorrhagic complications. Formal interaction analyses showed no significant effect modification by occlusion location across the evaluated outcomes. Accordingly, the location-specific patterns observed in descriptive analyses should be considered exploratory and hypothesis-generating rather than evidence of a location-dependent treatment effect. BGC use was also associated with higher odds of vasospasm, highlighting the need to balance potential procedural benefits against device-related vascular effects.

## Data Availability

The datasets presented in this study can be found in online repositories. The names of the repository/repositories and accession number(s) can be found in the article/Supplementary material.
