# Supplementary material for: Efficacy of balloon guide catheter in endovascular treatment according to occlusion location
Source: Front Neurol. 2026 Sep 30;17:1867575. doi: 10.3389/fneur.2026.1867575 (PMC13661195; doi:10.3389/fneur.2026.1867575)
Supplement: Supplementary file 1 [file Table_1.docx]

## OUTCOMES BY OCCLUSION LOCATION

**3.2.1 MCA-M1 (Proximal MCA) Occlusion**

In MCA-M1 occlusions, BGC use was associated with a significantly lower rate of distal embolism compared with non-BGC (52.6% vs. 70.1%; p=.010; χ²=6.704). No significant differences were observed in hemorrhage rates or vasospasm. The distribution of procedural techniques differed significantly between groups (p<.001): isolated stent-retriever use was markedly more common in the BGC group (53.5% vs. 8.2%), while the combined technique predominated in the non-BGC group (69.1% vs. 16.7%). FPR rates were comparable (48.2% BGC vs. 46.4% non-BGC; p=.788). Full results are presented in Table 3a.

**Table 3a. Outcomes by BGC Use in MCA-M1 Occlusions**

| **Variable** | **No BGC N (%)** | **BGC N (%)** | **P** | **χ²** |
| --- | --- | --- | --- | --- |
| Hemorrhage: Absent | 66 (68.0%) | 84 (73.7%) | .368 | .812 |
| Hemorrhage: Present | 31 (32.0%) | 30 (26.3%) |  |  |
| New territory embolsim: Absent | 29 (29.9%) | 54 (47.4%) | **.010*** | 6.704 |
| New territory embolsim: Present | 68 (70.1%) | 60 (52.6%) |  |  |
| Vasospasm: Absent | 66 (68.0%) | 68 (59.6%) | .207 | 1.593 |
| Vasospasm: Present | 31 (32.0%) | 46 (40.4%) |  |  |
| First-pass recanalization: No | 52 (53.6%) | 59 (51.8%) | .788 | .072 |
| First-pass recanalization: Yes | 45 (46.4%) | 55 (48.2%) |  |  |
| Technique: Aspiration | 22 (22.7%) | 34 (29.8%) | **.000*** | 69.151 |
| Technique: Combined | 67 (69.1%) | 19 (16.7%) |  |  |
| Technique: Isolated stent retriever | 8 (8.2%) | 61 (53.5%) |  |  |

** p<0.05. BGC, balloon guide catheter.* *ENT:Embolization to new territory*

**3.2.2 MCA-M2 (Distal MCA) Occlusion**

In MCA-M2 occlusions, there were no statistically significant differences in hemorrhage, distal embolism, or FPR rates between BGC and non-BGC groups. Notably, vasospasm was significantly more frequent in the BGC group (48.6% vs. 19.2%; p<.001; χ²=14.340). Again, procedural technique distribution differed significantly, with isolated stent retriever use predominating in the BGC group (64.3% vs. 14.1%), and the combined approach more common in the non-BGC group (62.8% vs. 15.7%). Full results are shown in Table 3b.

**Table 3b. Outcomes by BGC Use in MCA-M2 Occlusions**

| **Variable** | **No BGC N (%)** | **BGC N (%)** | **P** | **χ²** |
| --- | --- | --- | --- | --- |
| Hemorrhage: Absent | 58 (74.4%) | 48 (68.6%) | .436 | .608 |
| Hemorrhage: Present | 20 (25.6%) | 22 (31.4%) |  |  |
| New territory embolsim: Absent | 31 (39.7%) | 30 (42.9%) | .701 | .148 |
| New territory embolsim: Present | 47 (60.3%) | 40 (57.1%) |  |  |
| Vasospasm: Absent | 63 (80.8%) | 36 (51.4%) | **.000*** | 14.340 |
| Vasospasm: Present | 15 (19.2%) | 34 (48.6%) |  |  |
| First-pass recanalization: No | 29 (37.2%) | 28 (40.0%) | .725 | .124 |
| First-pass recanalization: Yes | 49 (62.8%) | 42 (60.0%) |  |  |
| Technique: Aspiration | 18 (23.1%) | 14 (20.0%) | **.000*** | 44.908 |
| Technique: Combined | 49 (62.8%) | 11 (15.7%) |  |  |
| Technique: Isolated stent retriever | 11 (14.1%) | 45 (64.3%) |  |  |

** p<0.05. BGC, balloon guide catheter.*

**3.2.3 ICA Occlusion**

In ICA occlusions, BGC use was associated with significantly lower rates of distal embolism (55.2% vs. 75.9%; p=.019; χ²=5.495) and significantly higher FPR (48.3% vs. 29.3%; p=.036; χ²=4.393). Hemorrhage and vasospasm rates were comparable between groups. The technique distribution differed markedly: combined technique was used in 69.0% of non-BGC cases and 32.8% of BGC cases, whereas aspiration was preferred in BGC-treated patients (43.1% vs. 29.3%). Full results are in Table 3c.

**Table 3c. Outcomes by BGC Use in ICA Occlusions**

| **Variable** | **No BGC N (%)** | **BGC N (%)** | **P** | **χ²** |
| --- | --- | --- | --- | --- |
| Hemorrhage: Absent | 41 (70.7%) | 44 (75.9%) | .529 | .396 |
| Hemorrhage: Present | 17 (29.3%) | 14 (24.1%) |  |  |
| New territory embolsim: Absent | 14 (24.1%) | 26 (44.8%) | **.019*** | 5.495 |
| New territory embolsim: Present | 44 (75.9%) | 32 (55.2%) |  |  |
| Vasospasm: Absent | 43 (74.1%) | 37 (63.8%) | .229 | 1.450 |
| Vasospasm: Present | 15 (25.9%) | 21 (36.2%) |  |  |
| First-pass recanalization: No | 41 (70.7%) | 30 (51.7%) | **.036*** | 4.393 |
| First-pass recanalization: Yes | 17 (29.3%) | 28 (48.3%) |  |  |
| Technique: Isolated stent retriever | 1 (1.7%) | 14 (24.1%) | **.000*** | 20.265 |
| Technique: Aspiration | 17 (29.3%) | 25 (43.1%) |  |  |
| Technique: Combined | 40 (69.0%) | 19 (32.8%) |  |  |

** p<0.05. BGC, balloon guide catheter.*

BGC use was independently associated with higher odds of functional independence at 90 days (adjusted OR [aOR], 1.613; 95% CI, 1.051–2.476; P=0.029). Younger age, lower baseline NIHSS, and collateral status were also independently associated with 90-day functional outcome (Supplementary Table S1).

**Supplementary Table S1. Multivariable logistic regression for 90-day functional independence (mRS 0-2) (n=473)**

| **Variable** | **β** | **SE** | **Adjusted OR** | **95% CI** | **P value** |
| --- | --- | --- | --- | --- | --- |
| BGC use | 0.478 | 0.219 | 1.61 | 1.05–2.47 | **0.029** |
| Occlusion location (overall) | — | — | — | — | 0.254 |
| Occlusion location (1) | -0.044 | 0.241 | 0.95 | 0.59–1.53 | 0.855 |
| Occlusion location (2) | -0.404 | 0.253 | 0.66 | 0.40–1.09 | 0.110 |
| Age | -0.023 | 0.009 | 0.97 | 0.96–0.99 | 0.007 |
| Sex | -0.142 | 0.204 | 0.86 | 0.58–1.29 | 0.486 |
| Baseline NIHSS | -0.093 | 0.022 | 0.91 | 0.87–0.95 | <0.001 |
| Collateral status | -0.440 | 0.211 | 0.64 | 0.42–0.97 | 0.037 |
| Atrial fibrillation | 0.113 | 0.219 | 1.12 | 0.72–1.72 | 0.606 |
| Hypertension | 0.337 | 0.222 | 1.40 | 0.90–2.16 | 0.128 |
| Type 2 diabetes mellitus | -0.216 | 0.254 | 0.80 | 0.49–1.32 | 0.394 |
| Coronary artery disease | 0.286 | 0.228 | 1.33 | 0.85–2.08 | 0.208 |

*Adjusted for BGC use, occlusion location, age, sex, baseline NIHSS, collateral status, atrial fibrillation, hypertension, type 2 diabetes mellitus, and coronary artery disease. OR, odds ratio; CI, confidence interval; SE, standard error; BGC, balloon guide catheter; NIHSS, National Institutes of Health Stroke Scale.*

BGC use was not independently associated with first-pass recanalization (aOR, 1.350; 95% CI, 0.893–2.043; P=0.155). Occlusion location was significant overall (P=0.001), and atrial fibrillation was independently associated with first-pass recanalization (aOR, 1.683; 95% CI, 1.120–2.529; P=0.012) (Supplementary Table S2).

Formun Altı

**Supplementary Table S2. Multivariable logistic regression for first-pass recanalization (n=473)**

| **Variable** | **β** | **SE** | **Adjusted OR** | **95% CI** | **P value** |
| --- | --- | --- | --- | --- | --- |
| BGC use | 0.300 | 0.211 | 1.35 | 0.8–2.043 | 0.155 |
| Occlusion location (overall) | — | — | — | — | 0.001 |
| Occlusion location (1) | 0.610 | 0.227 | 1.84 | 1.18–2.86 | 0.007 |
| Occlusion location (2) | -0.338 | 0.243 | 0.71 | 0.44–1.14 | 0.164 |
| Age | 0.008 | 0.008 | 1.00 | 0.99–1.02 | 0.288 |
| Sex | 0.160 | 0.193 | 1.17 | 0.80–1.71 | 0.407 |
| Baseline NIHSS | 0.011 | 0.021 | 1.01 | 0.97–1.05 | 0.589 |
| Collateral status | -0.398 | 0.204 | 0.67 | 0.45–1.00 | 0.051 |
| Atrial fibrillation | 0.521 | 0.208 | 1.68 | 1.12–2.52 | 0.012 |
| Hypertension | -0.251 | 0.210 | 0.77 | 0.51–1.17 | 0.232 |
| Type 2 diabetes mellitus | 0.069 | 0.241 | 1.07 | 0.66–1.71 | 0.775 |
| Coronary artery disease | 0.094 | 0.213 | 1.09 | 0.72–1.66 | 0.661 |

*Adjusted for BGC use, occlusion location, age, sex, baseline NIHSS, collateral status, atrial fibrillation, hypertension, type 2 diabetes mellitus, and coronary artery disease. OR, odds ratio; CI, confidence interval; SE, standard error; BGC, balloon guide catheter; NIHSS, National Institutes of Health Stroke Scale.*

BGC use was independently associated with lower odds of embolization to new territory (aOR, 0.536; 95% CI, 0.352–0.817; P=0.004). None of the other covariates reached statistical significance. (Supplementary Table S3).

Formun Üstü

**Supplementary Table S3. Multivariable logistic regression for embolization to new territory (n=473)**

| **Variable** | **β** | **SE** | **Adjusted OR** | **95% CI** | **P value** |
| --- | --- | --- | --- | --- | --- |
| BGC use | -0.623 | 0.215 | 0.53 | 0.35–0.81 | **0.004** |
| Occlusion location (overall) | — | — | — | — | 0.795 |
| Occlusion location (1) | -0.058 | 0.229 | 0.94 | 0.60–1.47 | 0.799 |
| Occlusion location (2) | 0.124 | 0.250 | 1.13 | 0.69–1.84 | 0.619 |
| Age | -0.003 | 0.008 | 0.99 | 0.98–1.01 | 0.719 |
| Sex | -0.031 | 0.196 | 0.96 | 0.66–1.42 | 0.874 |
| Baseline NIHSS | 0.015 | 0.021 | 1.01 | 0.97–1.05 | 0.480 |
| Collateral status | 0.198 | 0.209 | 1.21 | 0.8–1.836 | 0.343 |
| Atrial fibrillation | -0.380 | 0.209 | 0.68 | 0.45–1.02 | 0.068 |
| Hypertension | 0.179 | 0.213 | 1.19 | 0.78–1.81 | 0.400 |
| Type 2 diabetes mellitus | 0.253 | 0.249 | 1.28 | 0.79–2.09 | 0.310 |
| Coronary artery disease | -0.400 | 0.215 | 0.67 | 0.44–1.02 | 0.063 |

*Adjusted for BGC use, occlusion location, age, sex, baseline NIHSS, collateral status, atrial fibrillation, hypertension, type 2 diabetes mellitus, and coronary artery disease. OR, odds ratio; CI, confidence interval; SE, standard error; BGC, balloon guide catheter; NIHSS, National Institutes of Health Stroke Scale.*

BGC use was not independently associated with hemorrhagic complications (aOR, 1.148; 95% CI, 0.735–1.793; P=0.544). Increasing age was independently associated with higher odds of hemorrhagic complications (aOR per year, 1.022; 95% CI, 1.004–1.040; P=0.018). (Supplementary Table S4).

**Supplementary Table S4. Multivariable logistic regression for hemorrhagic complications (n=473)**

| **Variable** | **β** | **SE** | **Adjusted OR** | **95% CI** | **P value** |
| --- | --- | --- | --- | --- | --- |
| BGC use | 0.138 | 0.227 | 1.14 | 0.73–1.79 | 0.544 |
| Occlusion location (overall) | — | — | — | — | 0.917 |
| Occlusion location (1) | -0.053 | 0.244 | 0.94 | 0.58–1.53 | 0.829 |
| Occlusion location (2) | -0.108 | 0.264 | 0.89 | 0.53–1.50 | 0.683 |
| Age | 0.021 | 0.009 | 1.02 | 1.00–1.04 | 0.018 |
| Sex | -0.028 | 0.209 | 0.97 | 0.64–1.46 | 0.894 |
| Baseline NIHSS | -0.003 | 0.022 | 0.99 | 0.95–1.04 | 0.910 |
| Collateral status | -0.081 | 0.221 | 0.92 | 0.59–1.42 | 0.714 |
| Atrial fibrillation | 0.046 | 0.223 | 1.04 | 0.67–1.62 | 0.838 |
| Hypertension | 0.087 | 0.227 | 1.09 | 0.69–1.70 | 0.701 |
| Type 2 diabetes mellitus | 0.083 | 0.257 | 1.08 | 0.65–1.80 | 0.747 |
| Coronary artery disease | 0.208 | 0.227 | 1.23 | 0.78–1.92 | 0.360 |

*Adjusted for BGC use, occlusion location, age, sex, baseline NIHSS, collateral status, atrial fibrillation, hypertension, type 2 diabetes mellitus, and coronary artery disease. OR, odds ratio; CI, confidence interval; SE, standard error; BGC, balloon guide catheter; NIHSS, National Institutes of Health Stroke Scale.*

BGC use was independently associated with higher odds of vasospasm (aOR, 1.680; 95% CI, 1.092–2.584; P=0.018), whereas increasing age was associated with lower odds of vasospasm (aOR per year, 0.983; 95% CI, 0.967–0.998; P=0.031). (Supplementary Table S5).

**Supplementary Table S5. Multivariable logistic regression for vasospasm (n=473)**

| **Variable** | **β** | **SE** | **Adjusted OR** | **95% CI** | **P value** |
| --- | --- | --- | --- | --- | --- |
| BGC use | 0.519 | 0.220 | 1.68 | 1.09–2.58 | **0.018** |
| Occlusion location (overall) | — | — | — | — | 0.843 |
| Occlusion location (1) | -0.092 | 0.237 | 0.91 | 0.57–1.45 | 0.697 |
| Occlusion location (2) | -0.139 | 0.257 | 0.87 | 0.52–1.43 | 0.588 |
| Age | -0.018 | 0.008 | 0.98 | 0.96–0.99 | 0.031 |
| Sex | 0.152 | 0.202 | 1.16 | 0.78–1.72 | 0.453 |
| Baseline NIHSS | -0.001 | 0.021 | 0.99 | 0.95–1.04 | 0.958 |
| Collateral status | -0.194 | 0.216 | 0.82 | 0.53–1.25 | 0.370 |
| Atrial fibrillation | -0.216 | 0.219 | 0.80 | 0.52–1.23 | 0.324 |
| Hypertension | 0.272 | 0.219 | 1.31 | 0.85–2.01 | 0.215 |
| Type 2 diabetes mellitus | -0.255 | 0.255 | 0.77 | 0.47–1.27 | 0.317 |
| Coronary artery disease | 0.205 | 0.221 | 1.22 | 0.79–1.89 | 0.353 |

*Adjusted for BGC use, occlusion location, age, sex, baseline NIHSS, collateral status, atrial fibrillation, hypertension, type 2 diabetes mellitus, and coronary artery disease. OR, odds ratio; CI, confidence interval; SE, standard error; BGC, balloon guide catheter; NIHSS, National Institutes of Health Stroke Scale.*

**Supplementary Table S6. Multivariable interaction model for first-pass recanalization (n=473)**

| **Variable** | **β** | **SE** | **Adjusted OR** | **95% CI** | **P value** |
| --- | --- | --- | --- | --- | --- |
| BGC use (No vs Yes) | 0.882 | 0.409 | 2.42 | 1.08–5.39 | 0.031 |
| Occlusion: proximal MCA (M1) vs ICA | 0.004 | 0.332 | 1.00 | 0.52–1.92 | 0.991 |
| Occlusion: distal MCA (M2) vs ICA | -0.546 | 0.376 | 0.58 | 0.28–1.21 | 0.146 |
| Sex (0 vs 1) | 0.176 | 0.193 | 1.19 | 0.82–1.74 | 0.364 |
| Collateral status (0 vs 1) | -0.403 | 0.205 | 0.67 | 0.45–1.00 | 0.049 |
| Atrial fibrillation (No vs Yes) | 0.491 | 0.209 | 1.63 | 1.08–2.46 | 0.019 |
| Hypertension (No vs Yes) | -0.234 | 0.211 | 0.79 | 0.52–1.20 | 0.268 |
| Type 2 diabetes mellitus (No vs Yes) | 0.059 | 0.241 | 1.06 | 0.66–1.70 | 0.806 |
| Coronary artery disease (No vs Yes) | 0.080 | 0.214 | 1.08 | 0.71–1.65 | 0.708 |
| Age, per year | -0.009 | 0.008 | 0.99 | 0.98–1.01 | 0.266 |
| Baseline NIHSS, per point | -0.011 | 0.021 | 0.99 | 0.95–1.03 | 0.594 |
| BGC × proximal MCA (M1 vs ICA) | -0.720 | 0.487 | 0.49 | 0.19–1.26 | 0.140 |
| BGC × distal MCA (M2 vs ICA) | -0.815 | 0.530 | 0.44 | 0.16–1.25 | 0.124 |

*Note. Overall BGC × occlusion-location interaction: P=0.244. ICA was the reference category for occlusion location, and BGC use (Yes) was the reference category for BGC status. Interaction odds ratios quantify the difference in the estimated BGC effect for the specified MCA category relative to the ICA reference category. BGC, balloon guide catheter; MCA, middle cerebral artery; M1, proximal MCA; M2, distal MCA; ICA, internal carotid artery; NIHSS, National Institutes of Health Stroke Scale; OR, odds ratio; CI, confidence interval.*

**Supplementary Table S7. Multivariable interaction model for 90-day functional independence (n=473)**

| **Variable** | **β** | **SE** | **Adjusted OR** | **95% CI** | **P value** |
| --- | --- | --- | --- | --- | --- |
| BGC use (No vs Yes) | 0.321 | 0.409 | 1.38 | 0.62–3.07 | 0.433 |
| Occlusion: proximal MCA (M1) vs ICA | -0.658 | 0.355 | 0.52 | 0.26–1.04 | 0.064 |
| Occlusion: distal MCA (M2) vs ICA | -0.221 | 0.396 | 0.80 | 0.37–1.74 | 0.577 |
| Sex (0 vs 1) | -0.144 | 0.205 | 0.87 | 0.58–1.29 | 0.483 |
| Collateral status (0 vs 1) | -0.443 | 0.212 | 0.64 | 0.42–0.97 | 0.037 |
| Atrial fibrillation (No vs Yes) | 0.100 | 0.221 | 1.11 | 0.72–1.71 | 0.651 |
| Hypertension (No vs Yes) | 0.361 | 0.224 | 1.43 | 0.93–2.23 | 0.107 |
| Type 2 diabetes mellitus (No vs Yes) | -0.210 | 0.255 | 0.81 | 0.49–1.34 | 0.411 |
| Coronary artery disease (No vs Yes) | 0.297 | 0.228 | 1.35 | 0.86–2.11 | 0.193 |
| Age, per year | 0.023 | 0.009 | 1.02 | 1.01–1.04 | 0.009 |
| Baseline NIHSS, per point | 0.095 | 0.022 | 1.10 | 1.05–1.15 | <0.001 |
| BGC × proximal MCA (M1 vs ICA) | 0.524 | 0.505 | 1.69 | 0.63–4.54 | 0.299 |
| BGC × distal MCA (M2 vs ICA) | -0.206 | 0.543 | 0.81 | 0.28–2.36 | 0.704 |

*Note. Overall BGC × occlusion-location interaction: P=0.280. ICA was the reference category for occlusion location, and BGC use (Yes) was the reference category for BGC status. Interaction odds ratios quantify the difference in the estimated BGC effect for the specified MCA category relative to the ICA reference category. BGC, balloon guide catheter; MCA, middle cerebral artery; M1, proximal MCA; M2, distal MCA; ICA, internal carotid artery; NIHSS, National Institutes of Health Stroke Scale; OR, odds ratio; CI, confidence interval.*

**Supplementary Table S8. Multivariable interaction model for embolization to new territory (n=473)**

| **Variable** | **β** | **SE** | **Adjusted OR** | **95% CI** | **P value** |
| --- | --- | --- | --- | --- | --- |
| BGC use (No vs Yes) | -0.938 | 0.422 | 0.39 | 0.17–0.90 | 0.026 |
| Occlusion: proximal MCA (M1) vs ICA | 0.060 | 0.332 | 1.06 | 0.55–2.04 | 0.855 |
| Occlusion: distal MCA (M2) vs ICA | -0.195 | 0.375 | 0.82 | 0.39–1.72 | 0.603 |
| Sex (0 vs 1) | -0.039 | 0.197 | 0.96 | 0.65–1.41 | 0.842 |
| Collateral status (0 vs 1) | 0.200 | 0.210 | 1.22 | 0.81–1.84 | 0.340 |
| Atrial fibrillation (No vs Yes) | -0.349 | 0.210 | 0.71 | 0.47–1.06 | 0.096 |
| Hypertension (No vs Yes) | 0.155 | 0.214 | 1.17 | 0.77–1.78 | 0.469 |
| Type 2 diabetes mellitus (No vs Yes) | 0.254 | 0.250 | 1.29 | 0.79–2.10 | 0.310 |
| Coronary artery disease (No vs Yes) | -0.404 | 0.216 | 0.67 | 0.44–1.02 | 0.062 |
| Age, per year | 0.004 | 0.008 | 1.00 | 0.99–1.02 | 0.608 |
| Baseline NIHSS, per point | -0.017 | 0.021 | 0.98 | 0.94–1.03 | 0.431 |
| BGC × proximal MCA (M1 vs ICA) | 0.128 | 0.506 | 1.14 | 0.42–3.06 | 0.800 |
| BGC × distal MCA (M2 vs ICA) | 0.760 | 0.537 | 2.14 | 0.75–6.13 | 0.157 |

*Note. Overall BGC × occlusion-location interaction: P=0.267. ICA was the reference category for occlusion location, and BGC use (Yes) was the reference category for BGC status. Interaction odds ratios quantify the difference in the estimated BGC effect for the specified MCA category relative to the ICA reference category. BGC, balloon guide catheter; MCA, middle cerebral artery; M1, proximal MCA; M2, distal MCA; ICA, internal carotid artery; NIHSS, National Institutes of Health Stroke Scale; OR, odds ratio; CI, confidence interval.*

**Supplementary Table S9. Multivariable interaction model for hemorrhagic complications (n=473)**

| **Variable** | **β** | **SE** | **Adjusted OR** | **95% CI** | **P value** |
| --- | --- | --- | --- | --- | --- |
| BGC use (No vs Yes) | -0.051 | 0.439 | 0.95 | 0.40–2.25 | 0.908 |
| Occlusion: proximal MCA (M1) vs ICA | -0.144 | 0.380 | 0.87 | 0.41–1.82 | 0.705 |
| Occlusion: distal MCA (M2) vs ICA | -0.320 | 0.415 | 0.73 | 0.32–1.64 | 0.440 |
| Sex (0 vs 1) | -0.032 | 0.209 | 0.97 | 0.64–1.46 | 0.877 |
| Collateral status (0 vs 1) | -0.080 | 0.222 | 0.92 | 0.60–1.42 | 0.717 |
| Atrial fibrillation (No vs Yes) | 0.068 | 0.225 | 1.07 | 0.69–1.66 | 0.763 |
| Hypertension (No vs Yes) | 0.070 | 0.228 | 1.07 | 0.69–1.68 | 0.758 |
| Type 2 diabetes mellitus (No vs Yes) | 0.082 | 0.258 | 1.09 | 0.65–1.80 | 0.751 |
| Coronary artery disease (No vs Yes) | 0.208 | 0.228 | 1.23 | 0.79–1.92 | 0.361 |
| Age, per year | -0.021 | 0.009 | 0.98 | 0.96–1.00 | 0.023 |
| Baseline NIHSS, per point | 0.001 | 0.022 | 1.00 | 0.96–1.05 | 0.950 |
| BGC × proximal MCA (M1 vs ICA) | 0.055 | 0.526 | 1.06 | 0.38–2.96 | 0.916 |
| BGC × distal MCA (M2 vs ICA) | 0.504 | 0.568 | 1.66 | 0.54–5.04 | 0.375 |

*Note. Overall BGC × occlusion-location interaction: P=0.579. ICA was the reference category for occlusion location, and BGC use (Yes) was the reference category for BGC status. Interaction odds ratios quantify the difference in the estimated BGC effect for the specified MCA category relative to the ICA reference category. BGC, balloon guide catheter; MCA, middle cerebral artery; M1, proximal MCA; M2, distal MCA; ICA, internal carotid artery; NIHSS, National Institutes of Health Stroke Scale; OR, odds ratio; CI, confidence interval.*

**Supplementary Table S10. Multivariable interaction model for vasospasm (n=473)**

| **Variable** | **β** | **SE** | **Adjusted OR** | **95% CI** | **P value** |
| --- | --- | --- | --- | --- | --- |
| BGC use (No vs Yes) | 0.279 | 0.422 | 1.32 | 0.58–3.02 | 0.510 |
| Occlusion: proximal MCA (M1) vs ICA | -0.087 | 0.342 | 0.92 | 0.47–1.79 | 0.799 |
| Occlusion: distal MCA (M2) vs ICA | -0.488 | 0.380 | 0.61 | 0.29–1.29 | 0.200 |
| Sex (0 vs 1) | 0.148 | 0.203 | 1.16 | 0.78–1.73 | 0.466 |
| Collateral status (0 vs 1) | -0.199 | 0.217 | 0.82 | 0.54–1.25 | 0.360 |
| Atrial fibrillation (No vs Yes) | -0.176 | 0.222 | 0.84 | 0.54–1.29 | 0.426 |
| Hypertension (No vs Yes) | 0.241 | 0.221 | 1.27 | 0.83–1.96 | 0.275 |
| Type 2 diabetes mellitus (No vs Yes) | -0.266 | 0.256 | 0.77 | 0.46–1.27 | 0.299 |
| Coronary artery disease (No vs Yes) | 0.199 | 0.223 | 1.22 | 0.79–1.89 | 0.371 |
| Age, per year | 0.020 | 0.008 | 1.02 | 1.00–1.04 | 0.017 |
| Baseline NIHSS, per point | -0.002 | 0.021 | 1.00 | 0.96–1.04 | 0.927 |
| BGC × proximal MCA (M1 vs ICA) | -0.144 | 0.507 | 0.87 | 0.32–2.34 | 0.777 |
| BGC × distal MCA (M2 vs ICA) | 0.964 | 0.564 | 2.62 | 0.87–7.92 | 0.087 |

*Note. Overall BGC × occlusion-location interaction: P=0.063. ICA was the reference category for occlusion location, and BGC use (Yes) was the reference category for BGC status. Interaction odds ratios quantify the difference in the estimated BGC effect for the specified MCA category relative to the ICA reference category. BGC, balloon guide catheter; MCA, middle cerebral artery; M1, proximal MCA; M2, distal MCA; ICA, internal carotid artery; NIHSS, National Institutes of Health Stroke Scale; OR, odds ratio; CI, confidence interval.*

## ****Supplementary Table S11** -**Ordinal logistic regression analysis of 90-day modified Rankin Scale (shift analysis)

Model adjusted for balloon guide catheter use, age, sex, baseline NIHSS, collateral status, and occlusion location.

| Variable | β | SE | Common OR | 95% CI | P |
| --- | --- | --- | --- | --- | --- |
| Balloon guide catheter use | 0.400 | 0.180 | 1.49 | 1.05–2.12 | **0.026** |
| Age (per year) | 0.021 | 0.007 | 1.02 | 1.01–1.03 | **0.002** |
| Baseline NIHSS | 0.089 | 0.018 | 1.09 | 1.06–1.13 | **<0.001** |
| Poor collateral status | -0.451 | 0.175 | 0.64 | 0.45–0.90 | **0.010** |
| Sex | -0.234 | 0.167 | 0.79 | 0.57–1.10 | 0.161 |
| MCA proximal vs ICA | -0.279 | 0.208 | 0.76 | 0.50–1.14 | 0.179 |
| MCA distal vs ICA | -0.094 | 0.228 | 0.91 | 0.58–1.42 | 0.679 |

### Model performance

• Likelihood ratio χ² = 79.262, df = 7, p < 0.001.

• Test of Parallel Lines: χ² = 39.486, df = 35, p = 0.276 (proportional odds assumption satisfied).
